# Metaproteomics to understand how microbiota function: The crystal ball predicts a promising future

**DOI:** 10.1111/1462-2920.16238

**Published:** 2022-10-18

**Authors:** Jean Armengaud

**Affiliations:** ^1^ Département Médicaments et Technologies pour la Santé (DMTS) Université Paris‐Saclay, CEA, INRAE Bagnols‐sur‐Cèze France

## Abstract

In the medical, environmental, and biotechnological fields, microbial communities have attracted much attention due to their roles and numerous possible applications. The study of these communities is challenging due to their diversity and complexity. Innovative methods are needed to identify the taxonomic components of individual microbiota, their changes over time, and to determine how microoorganisms interact and function. Metaproteomics is based on the identification and quantification of proteins, and can potentially provide this full picture. Due to the wide molecular panorama and functional insights it provides, metaproteomics is gaining momentum in microbiome and holobiont research. Its full potential should be unleashed in the coming years with progress in speed and cost of analyses. In this exploratory crystal ball exercise, I discuss the technical and conceptual advances in metaproteomics that I expect to drive innovative research over the next few years in microbiology. I also debate the concepts of ‘microbial dark matter’ and ‘Metaproteomics‐Assembled Proteomes (MAPs)’ and present some long‐term prospects for metaproteomics in clinical diagnostics and personalized medicine, environmental monitoring, agriculture, and biotechnology.

## INTRODUCTION

Although most of our current knowledge comes from isolates, the role of microorganisms cannot be fully understood when analysed individually. Indeed, in nature, microorganisms generally exist within the framework of more complex biological systems. Therefore, they are challenging to study, even with the most advanced techniques. In this review, I will focus on one of these advanced techniques: metaproteomics, an emerging methodology with several facets that may not all be familiar to most readers. For a more detailed overview of the history and analytical process, I recommend several key articles of general interest (Hettich et al., 2012; Hettich et al., 2013; Salvato et al., 2021) and reviews devoted to data analysis (Heyer et al., 2017; Muth et al., 2016; Sajulga et al., 2020). Here, I will highlight the specificities of the approach when used to taxonomically and functionally characterize microbiota, and discuss current associated issues and long‐term perspectives for applications.

In January 1995, having completed my Ph.D. on the characterization of molecular mechanisms behind the electron transport in a photosynthetic microorganism, I engaged in a rich post‐doctoral experience by joining the laboratory of Professor Kenneth Nigel Timmis in Braunschweig, Germany. Over the subsequent 4 years under his guidance, I absorbed knowledge of microorganisms and microbial communities, and contributed to the description of the various dioxin‐degradative enzymes of the bacterium *Rhizorhabdus wittichii* RW1 (formerly *Sphingomonas wittichii* RW1), aiming at identifying new catalysts that could be useful for the bioremediation of polluted environments (Armengaud et al., 1998). At the time, a technological revolution was underway in the form of the rise of high‐throughput DNA sequencing. This revolution led to (i) the advent of 16S rRNA gene amplicon sequencing and its application to characterize the structure of microbial communities, and (ii) the first use of shotgun genomics strategies to sequence large DNA fragments. In parallel, proteomics was emerging as a promising technique (Roepstorff, 1997). Back then, it involved two‐dimensional denaturing gel electrophoresis, extensive pattern comparison, and mass spectrometry analysis of the most interesting protein spots. In the lab, we applied both approaches to describe key molecular players of the studied microorganisms. In a friendly and highly collaborative setting created, powered, and managed by Ken, and energized by many scientists and visitors from around the world, we learned Science and gained insights into the key biological questions that deserve priority investigation. This experience set the stage for the next steps in my scientific career, which led me to focus on proteomics through all its iterations.

This crystal ball review on metaproteomics is my humble tribute of thanks to my much‐appreciated colleague and friend Professor Kenneth Nigel Timmis. His life's work has made him an outstanding microbiologist who has inspired many researchers. He founded and was the long‐time Editor‐in‐Chief of the scientific journals ‘Environmental Microbiology’ and ‘Environmental Microbiology Reports’, two central arenas for disseminating outstanding research findings and ideas (Timmis, 2022; Timmis & Timmis, 2018).

## METAPROTEOMICS, MUCH MORE THAN JUST ADDING ANOTHER META‐OMICS METHODOLOGY

Omics methodologies are classically presented as individualized silos to make it easier for the audience to understand the underlying concepts and applications. However, this naïve presentation tends to limn the different approaches as mutually exclusive, whereas the links and synergies between omics strategies should be emphasized. For example, high‐quality genome sequencing, optimized assembly, and improved genome annotation are required to perform high‐quality proteomics analysis, as the latter is based on a theoretical database built with protein sequences derived from the annotated genome. Missing genomic sequences will lead to protein misinterpretations. In turn, proteomics results are invaluable when seeking to refine genome annotation or assess its quality according to orthogonal parameters (Armengaud, 2009). Similarly, metabolomics and proteomics are intricately linked. Identification and quantification of enzymes can only provide so much information on the metabolic processes deployed by an organism, the picture can be completed with precise quantification of the metabolites they produce. The proteome can then be used to elucidate the various routes and fates of these metabolites. Studying microbiota with the full range of omics is the key to success (Heintz‐Buschart & Wilmes, 2018).

Metagenomics is the study of metadata acquired by sequencing the total DNA extracted from a sample containing multiple organisms. By analogy, metaproteomics identifies and quantifies proteins derived from complex samples containing various microorganisms and possibly host cells (Wilmes & Bond, 2006). The proteins being considered as the workhorses of biological systems, their study highlights the players responsible for conducting specific functions, participating as building blocks, and coordinating the biological processes. The methodology is directly derived from classical single‐organism proteomics, involving protein extraction, trypsin proteolysis, and analysis of the resulting peptides by high‐resolution tandem mass spectrometry coupled with reverse‐phase chromatography. Data are queried against a protein sequence database to identify the peptide sequences, and quantitative values are assigned to them (Gouveia, Grenga, et al., 2020). Unlike when analysing nucleic acid molecules by the polymerase chain reaction, this process involves no amplification step. Thus, peptides are directly monitored by mass spectrometry, and consequently, the measurement is, in principle, unbiased. The results for a given peptide can be directly compared between distinct samples. However, the signal from individual molecules is not inherently equal because each molecule has its ionization characteristics, and other signals can interfere with its measurement. Nonetheless, the average resulting behaviour will be roughly comparable, and consequently, many signals can be merged and compared. Proteins are identified by their peptides in single‐organism proteomics, but the multiple combinations that may occur in the database used for the interpretation of data obtained from complex samples make this identification exceedingly tricky.

To circumvent this difficulty, the concept of protein groups has taken on its full value in metaproteomics. Protein groups are formed based on peptides shared between group members, and sometimes unambiguous peptides, that is, unique in the whole database, after applying parsimony rules—‘the simplest explanation or solution is the best one’. Then, the abundances of these groups can be compared between samples. Interpretation of results can be usefully guided by any prior information available on the sample. For example, metataxonomics‐derived information can be used to design the most appropriate protein sequence database focusing only on the organisms present in the sample. Ideally, metagenomics data or a sample‐specific mega‐catalogue of genes can be used to produce the protein sequence database to target the most likely events (Tanca et al., 2013). However, the use of such giant databases complicates the interpretation of MS/MS spectra, and more particularly the evaluation of their degree of confidence. It is therefore necessary to reduce the database to the organisms actually present in abundance in the sample. Strategies involving sectioning and database enrichment (Kumar et al., 2020) or a cascade of successive searches in order to reduce the search space are particularly suitable (Jouffret et al., 2021). It should be noted that, because the peptide sequences identified by metaproteomics include direct taxonomic information, identifying the taxa contained in a given microbiota has become a straightforward result of metaproteomics (Hardouin et al., 2021; Mesuere et al., 2018). The procedure involves tandem mass spectrometry‐proteotyping microorganisms by identifying a myriad of peptide sequences, some of which are specific to a particular branch of the Tree of Life, whereas others are common to several distinct but phylogenetically related taxonomic groups. Yet others are less informative because they are randomly shared across many organisms. Remarkably, this approach applies to all branches of the Tree of Life, resulting in a broad view of the organisms present in the sample: archaea, fungi, yeasts, algae, parasites, bacteria, and even animals and plants can be treated equally, provided the method applied for protein extraction works similarly well for them all. Thus, today, proteotyping based on metaproteomics is a powerful means to assess the structure of microbial communities. The results agree with those obtained by other molecular approaches (Jouffret et al., 2021; Van Den Bossche, Kunath, et al., 2021). Interestingly, in principle, no prior information about the sample is needed, which reduces the analysis costs per sample. This is well exemplified by the metaproteomics exploration of the gut microbiome of a millimetric amphipod used as a sentinel of aquatic environments for which no prior results obtained by metagenomics or metabarcoding of the microbiota was necessary (Gouveia, Pible, et al., 2020). In this study on minute amount of biological material, the quantitative values associated with the identified peptides were used to estimate each taxon's respective protein biomass ratio, thus harmonizing the quantitative parameters regardless of the taxon considered.

The most important result of the metaproteomics analysis is the information on which microorganisms are functioning and how, provided by the proteins detected (Van Den Bossche, Arntzen, et al., 2021). This functioning is described by two variables: the proteins corresponding to functional units and their abundance, which provides a proxy for their activity. Furthermore, the metaproteomics methodology applied to the holobiont can also offer a unique functional characterization of the hosts' molecular response (Grenga et al., 2022; Heintz‐Buschart & Wilmes, 2018). Focusing on the proteins secreted or released by cells by analysing the ‘exoproteome’ can shed light on how the cellular units of the microbiota interact with each other and with their environment (Lidbury et al., 2022; Xie et al., 2022). In most cases, the peptides identified can then be used to trace the specific organisms that produced the corresponding proteins, allowing an accurate molecular description of their phenotype, at least for the most abundant taxa. However, the list of proteins identified and their abundance in the sample does not directly reveal the functioning of the biological system, as expert biological knowledge is required. Interestingly, curated databases including information on the relationships between microorganisms, metabolites, and proteins are proposed (Cheng et al., 2022). In the future, integrative tools that provide an overview of metabolic pathways for the tens of thousands of proteins identified and characterize how the microbial machinery interacts and functions are awaited. At last, comparative metaproteomics performed on dissimilar samples appears to be the most appropriate approach to apply, as sample comparison can highlight the most distinct key elements explaining the observed phenotypes.

## CURRENT MAJOR CHALLENGES IN METAPROTEOMICS

**FIGURE 1:**
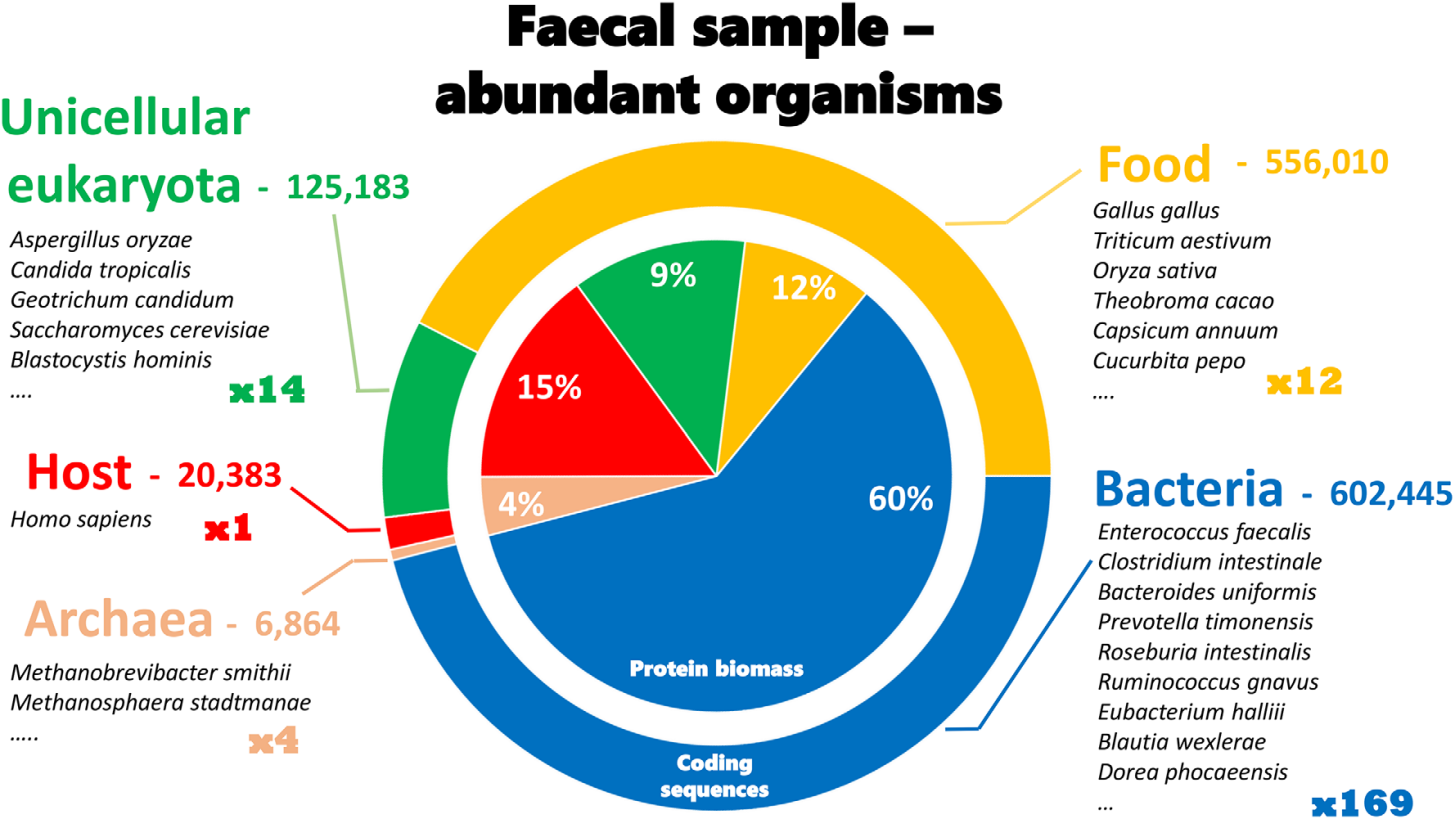
Estimation of the molecular complexity of a human faecal sample. The numbers indicated are derived from published results (Grenga et al., 2022), obtained from 39 human faecal samples analysed in triplicate. The different groups of identified organisms are indicated with the number of entities (×1 for the host) and a list of representative species per group is mentioned. The number of annotated protein‐coding sequences per group (20,383 for the host) is mentioned and their ratios are represented with the external circle. The inner circle shows the protein biomass of each group assessed experimentally by metaproteomics.

**FIGURE 2:**
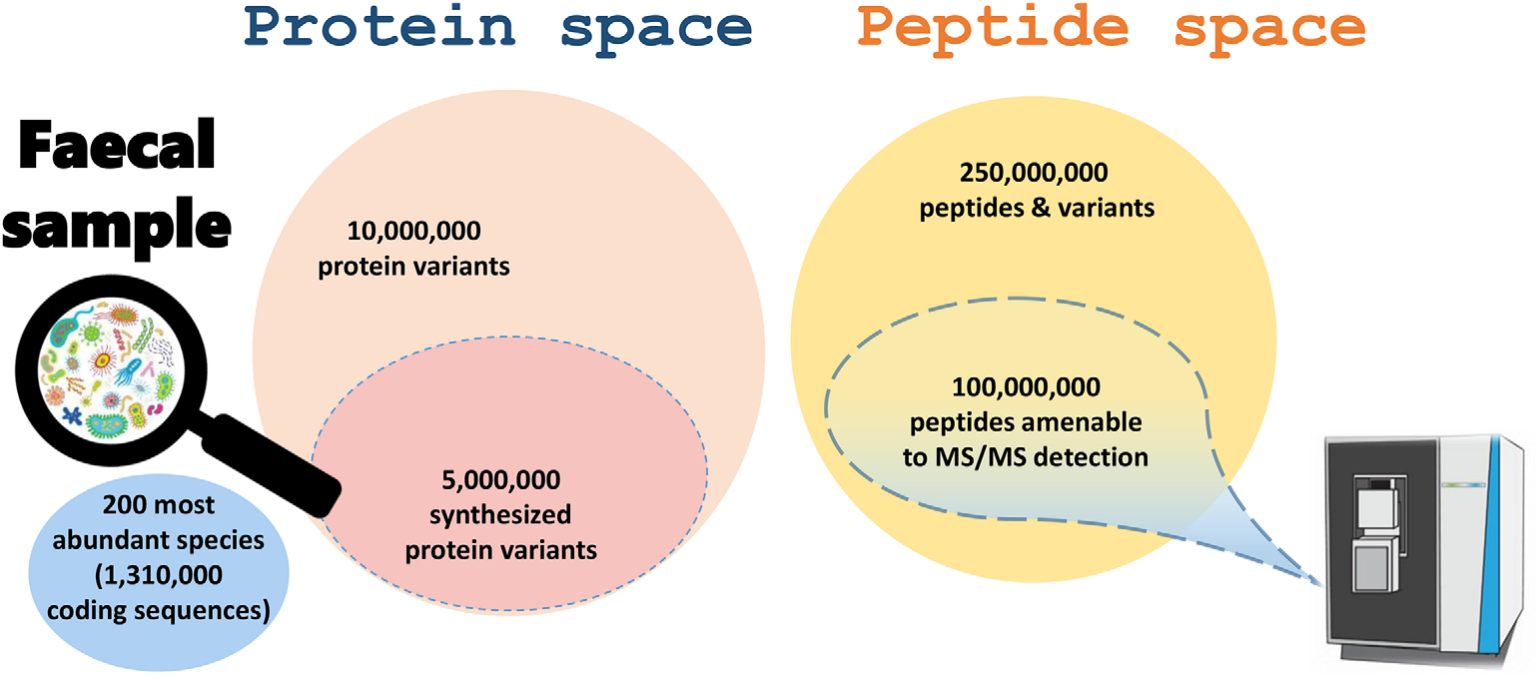
Tentative estimation of the protein and peptide spaces of a human faecal sample. The protein space is directly derived from the number of organisms estimated in Figure 1 and focussed on the most abundant organisms. Peptides are considered with equate I/L as these two residues are indistinguishable by simple mass spectrometry, with an average of 24 peptides per protein without missed‐cleavage and 70 peptides per protein when considering 1 possible missed‐cleavage. An average of 5 variants per peptide sequence is taken into account for synthesized proteins from the 200 most abundant species to obtain the number of peptides & variants.

If we take a representative sample of the human gastrointestinal tract as a reference (Rajilic‐Stojanovic et al., 2007), among its most abundant components we can list more than a hundred species of bacteria, a few archaea, dozens of species of fungi and yeasts, perhaps one or more parasites such as the common *Blastocystis*, a range of plant‐ and animal‐derived food residues, and host proteins. Figure 1 shows a representation of such a sample, with the number of organisms per category, the number of protein coding sequences deduced from their genome annotation, and an experimental estimation of their respective biomasses assessed by metaproteomics of faecal samples (Grenga et al., 2022). As observed in Figure 1, bacteria contribute the most abundant protein biomass, but host, food, and other eukaryotes are far to be negligible. It is evident from this figure that the interpretation of such metaproteomics data should not be biased with a restricted subset of the database corresponding only to bacteria. The pan‐genome of any such a sample will likely comprise more than 22 Gb of nucleotides, potentially coding for well over 1.3 million possible polypeptides. Because the entities present are not just single representatives of each species, but a broad compilation of different strains, the number of polypeptides may be multiplied many times (Figure 2), leading to a crowd of variants. The resulting metaproteomics‐derived estimation of the most abundant components fits well with the database size of the Integrated nonredundant Gene Catalogue constructed by extensive metagenomics of the human gut microbiome and comprising 9,879,896 genes (Li et al., 2014). However, only half of the potential coding sequences are probably translated into final products, as some genes might be silenced under specific harvesting conditions. This effect will somehow reduce the final number. Nevertheless, the number remains very large compared to those resulting from proteomics analysis performed on human cell lines, where at best less than 1% of that number is managed in the initial extract. Post‐translational modifications of proteins can complexify the picture further through the introduction of multiple proteoforms (den Ridder et al., 2020). The resulting peptide space is illustrated in Figure 2. When treated with a protease such as trypsin and—in line with standard practice—allowing a single missed proteolytic cleavage, the number of possible peptide sequences present in the mixture to be analysed can be roughly estimated at around a quarter of billion. However, less than 40% of these peptides are potentially amenable to tandem mass spectrometry measurements, that is, 100 million entities. Admittedly, this estimate is far from reality, but it sets the scene. Such a huge number poses three daunting challenges: (i) even the most advanced analytical system is not fast enough to identify all of these peptides, and the tandem mass spectrometer will randomly sample the most abundant peptides contained in the tube to be analysed, (ii) peptides with identical hydrophobicity characteristics and closely related molecular masses will frequently co‐elute; as a result, a large number of peptides will be co‐fragmented, making the interpretation of the resulting scrambled tandem mass spectrometry spectra difficult, and (iii) the absolute amount of each peptide eluted from the chromatographic system will be lower than what would be available during single‐species proteomics measurements because the total quantity of peptide material injected onto the reverse‐phase chromatography column is generally kept constant due to physical constraints. The great diversity of entities to be measured by mass spectrometry must also be considered alongside their respective abundances. The current generation of mass spectrometers has a relatively limited dynamic range of measurements, meaning that only the most abundant peptides will be sought. Consequently, it is impossible to probe the full dynamic range of peptides. Ultimately, although the same analytical system is used for single‐species proteomics and metaproteomics, the two approaches are very different when considering peptide diversity and dynamic range. As explained above, the peptide diversity contained in metaproteomics samples is enormous, and the analytical system can only sample part of this diversity (Lohmann et al., 2020). Devoting more mass spectrometry measurement time to each sample should logically increase the number of identified peptides, but are the efforts worth it when the asymptotic increase rapidly proves costly for a non‐significant gain? Currently, a comprehensive metaproteome is therefore simply unattainable. Depending on the biological system studied, scratching the surface may not be sufficient to glean insightful biological information. Nevertheless, careful evaluation of the results and investigation of the saturation effect are useful to verify the depth of analysis required to characterize the keystone species. Metaproteomics may be limited in sensitivity but has the advantage of focusing on the most significant molecular events. Indeed, a recent benchmark analysis of reference samples conducted by the metaproteomics community showed that almost identical pictures were obtained both in terms of taxonomy and functions using distinct analytical platforms and setups, suggesting that current mass spectrometry restrictions on the precursor sampling are not such an issue for well‐equipped platforms, and staking a claim for the maturity of the methodology (Van Den Bossche, Kunath, et al., 2021).

A series of emerging innovations in mass spectrometry are revolutionizing single‐species proteomics today and could logically and advantageously be applied in metaproteomics in the future. First, the introduction of ion mobility as an additional means to filter or analyse ions can serve to further fractionate peptide mixtures, thus limiting peptide co‐elution. In addition, the data‐independent acquisition mode has recently been generalized to allow a larger number of signals to be recorded (Kitata et al., 2022). Data‐dependent acquisition deals with ions from a single peptide: isolating, aggregating, and then fragmenting them in the mass spectrometer to generate a simple tandem mass spectrum that can be confidently assigned. In contrast, data‐independent acquisition is based on energy‐induced fragmentation of several peptides simultaneously to produce complex tandem mass spectra. This new acquisition mode identifies several peptides in chorus, but with less confidence. Its pioneering application to metaproteomics has been reported for the analysis of gut microbiota by two independent groups (Aakko et al., 2020; Long et al., 2020), but additional validation with representative microbiota samples would be welcome. With such approaches, pipelines to handle the giant databases typically used in metaproteomics and to interpret complex data‐independent acquisition will need to be optimized and carefully benchmarked.

Another significant gain could be provided by using new reverse‐phase liquid chromatography columns with improved performance to allow the resolution of closely related entities. Longer reverse‐phase columns with increased pore size or improved surface structure must be operated at higher pressure, requiring higher performance chromatography systems.

Finally, a new generation of high‐resolution tandem mass spectrometers with improved dynamic range, sensitivity, and acquisition speed would be more than welcome for metaproteomics applications. In addition, the various operations (i.e., sample preparation possibly including multiplexing, peptide chromatography and mass spectrometry, and interpretation) must be made more robust, at a lower cost, and further developed if we wish the methodology to be adopted by a greater number of users.

These materials and methodological advances will undoubtedly provide significant improvements, but the most decisive challenges lie in the interpretation of the data acquired (Schiebenhoefer et al., 2019). As a first step to improve the methodology for all users, a series of dedicated databases could be constructed, shared, and regularly refined to better reflect the diversity and respective abundances of the taxonomical units present in the most common samples. Such comprehensive gene catalogues established with high‐quality shotgun metagenomics data and pan‐genomics surveys have been successfully used for metaproteomics studies of human gut samples (Bassignani et al., 2021) and soil samples (Jouffret et al., 2021). To avoid misinterpretation, these databases should ideally only include well‐assembled and annotated genomes and curated taxonomical data (Pible & Armengaud, 2015). Efforts to refine methods for peptide identification and protein inference in the context of giant databases and highly redundant data are currently drawing a lot of fire‐power (Muth et al., 2016). Logically, improvements to the functional annotation of polypeptides should add considerable value to any metaproteomics analysis (de Crecy‐Lagard et al., 2022). Current pipelines based on Gene Ontology (GO) term analysis or KEGG annotation are surprisingly informative when analysing differences between samples in terms of function and activity (Verschaffelt et al., 2021; Walke et al., 2021). This information could potentially be refined by adding conserved structural protein domains, protein/protein interaction searches, or machine learning associated features based on massive data. Finally, functional information on the biological context and the specific condition of the sample must be integrated. However, to make these improvements, our representation of the microbiome may need to be revised, along with our view of its overall metabolism and the various metabolic pathways used by the most abundant taxonomical components.

Another major recent innovation in proteomics is single‐cell proteomics, whereby the heterogeneity of a population of cells can be measured, providing a new dimension of knowledge (Ctortecka et al., 2022). The throughput of such single‐cell analysis in data‐independent acquisition mode can be advantageously increased by multiplexing thanks to non‐isobaric mass tags (Derks et al., 2022). Given the sensitivity of current tandem mass spectrometers, and that metaproteomics aims to analyse microbial organisms that are a thousand times smaller than the volume of a mammalian cell, single‐cell metaproteomics appears out of reach for the moment. However, why not ask mass spectrometer manufacturers to design an instrument that could achieve the sensitivity needed for a single microorganism, or at least allow low input‐level measurements and dream a little about spatial metaproteomics scans? With another take on the individuality concept, I am convinced that isolating and culturing representatives of organisms from an uncharacterized branch of life is worthwhile from the moment a microbiome study highlights their possible presence in the sample. Obtaining such isolates, or even reduced consortia enriched in these microorganisms, would make it possible to confirm meta‐omics observations and envisage more mechanistic experiments to highlight their characteristics and better define their potential keystone role. In the next decade, we will potentially see many combinations of culturomics‐oriented projects with microbiota meta‐omics studies.

## REVISITING IMPORTANT CONCEPTUAL PARADIGMS

**FIGURE 3:**
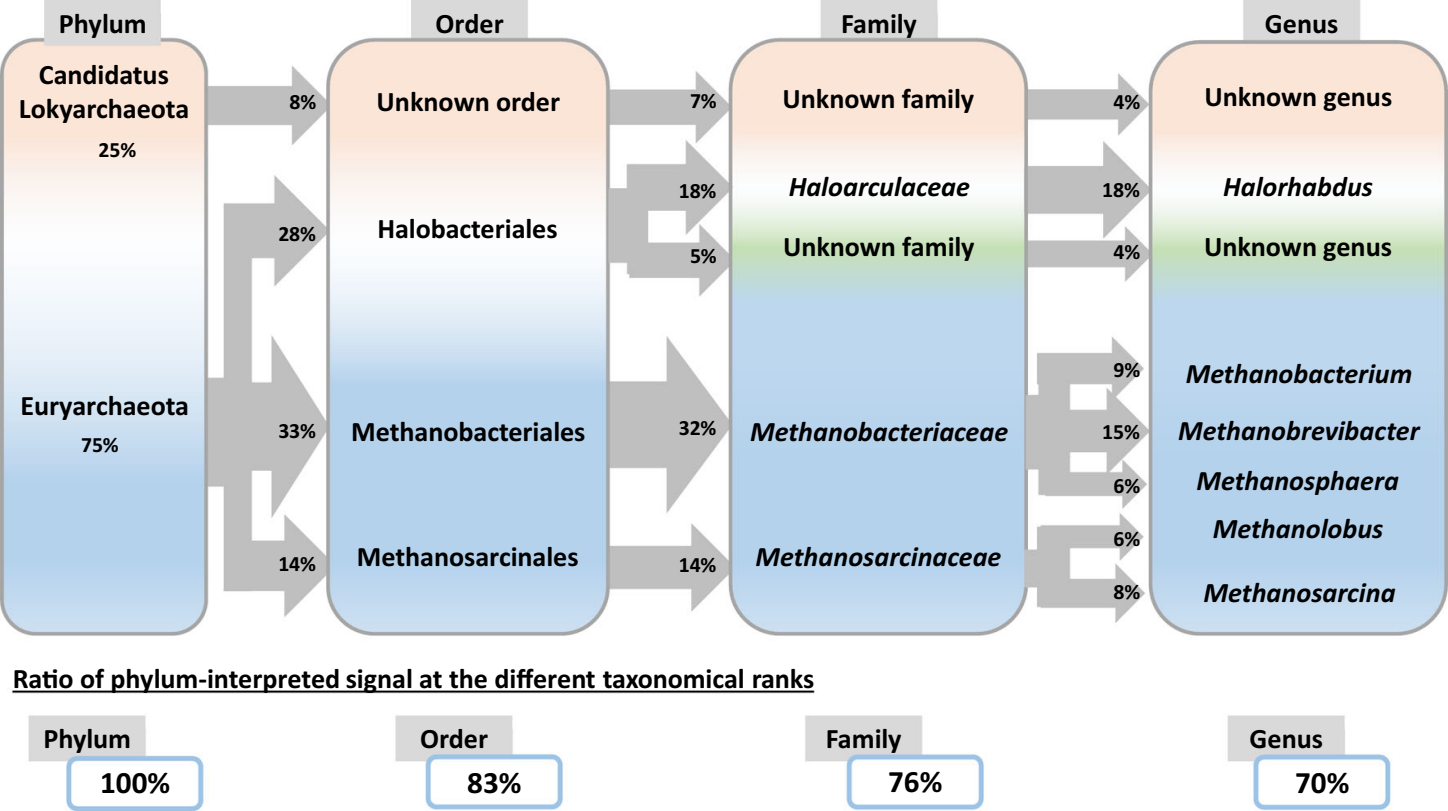
Metaproteomics can identify and quantify uncharted branches of the tree of life. Tandem mass spectrometry‐based proteotyping of organisms present in the sample can be based on taxa‐spectrum matches (TSMs) and taxon‐specific peptides (Hardouin et al., 2022; Lozano et al., 2022). In this figure focused on a theoretical archaeal enriched microbial community, the presence of an uncharacterized organism belonging to the Candidatus Lokyarchaeota phylum and another belonging to the Halobacteriales order are indicated, while other organisms characterized at the genus taxonomical rank are also confirmed. These organisms are identified from taxon‐specific peptides and TSMs at the different taxonomical ranks when querying a generalist database such as NCBInr. The ratios of organisms are established based on protein biomass values derived from the TSMs parameter and are reported in percentage compared to the signal interpreted for the phylum taxonomical rank. The values presented are imaginary and serve only to explain the concept. A decrease of TSMs along the taxonomical ranks may be observed due to the phylogenetic distance between the proteins from the organisms present in the sample and those from the organisms listed in the database.

Several years ago, I heard about ‘microbial dark matter’ to describe that part of the sample corresponding to uncharted branches of the Tree of Life. The name was an attempt at a parallel with the astronomic ‘dark matter’—the non‐luminous material thought to exist but for which we lack firm experimental evidence. However, significant differences exist. Unravelling the microbial dark matter is limited by the lack of sequenced genomes for uncharacterised microorganisms (Marcy et al., 2007; Rinke et al., 2013). Some authors even proposed that microorganisms that have not yet been *cultured* to be included in the ‘microbial dark matter’ (Vollmers et al., 2022). Despite the traumatic shockwave this may trigger, I am convinced that the ‘microbial dark matter’ does not exist as such, and the very concept is biased. This is because of a taxonomic peculiarity provided by metaproteomics that many ignore. Even uncharacterized microorganisms can now be fairly well taxonomically characterized from even small amounts of genomic information (Murray et al., 2021). Nowadays, genome sequences, well‐annotated metagenome‐assembled genomes (MAGs), or single amplification genomes (SAGs) are available for at least a few representatives of most phyla. Thanks to this information, proteotyping based on metaproteomics can give the full panorama of the taxa present in any sample, at least at the highest taxonomic ranks. As illustrated in Figure 3, many phylum‐specific peptides can be used to determine which phyla are present in samples and their respective ratios. At a lower taxonomic rank, certain classes, orders, or families may be under‐represented in, or simply absent from, the database due to a lack of representative genomes. For these branches of life, metaproteomics cannot provide fine taxonomic and functional information. Still, it will help to delineate which branch of the Tree of Life (phylum, class, order, family, genus) has been refined as far as currently possible and which ones are missing but explain the signal observed at the higher taxonomic ranks. Thus, rather than representing ‘microbial dark matter’, these specific components of microbial communities can, in principle, be differentiated by connecting them to a higher taxonomic rank, and consequently they can be quantified. Naturally, this metaproteomics‐derived information can help prioritize further taxonomic studies to improve our knowledge of these specific taxa, to shed more and more light on the darkness! The scope of this concept must be argued with specific examples, and tools will need to be developed to explore its applications.

By analogy with MAGs constructed from deep, high‐quality metagenomic data (Lee et al., 2017), the possibility of reconstructing metaproteomics‐assembled proteomes, or ‘MAPs’, could be envisioned. This collected information could be used to understand the physiology of the corresponding yet uncultured organism and establish its position in the Tree of Life. Whenever a new uncharacterized branch of life is detected by metaproteomics thanks to informative taxon‐specific peptides at a high taxonomic rank, it should be conceivable to identify its proteins. This would be made possible by identifying the most conserved protein sequences of this hitherto uncharacterized organism, even if the database does not yet contain information about its genome. They can, in principle, be assigned to identified taxa by applying parsimony rules based on the exact amounts of each taxon that could be established by the recently‐introduced concept of phylopeptidomics (Pible et al., 2020). This truly ground‐breaking methodology is based on mathematical modelling of the experimental peptide signals shared across all the organisms present in the database queried for the interpretation. Less conserved sequences of these MAPs that would point at the most specific traits of these organisms could benefit from de novo sequencing of unassigned tandem mass spectrometry spectra. This procedure has already been shown to be applicable in metaproteomics (Kleikamp et al., 2021). Tools for the intelligent reconstruction of protein sequences could be guided by the impressive amount of well‐established protein sequences we already have. Admittedly, orchestrating such MAPs will be far from trivial, as it will involve ultra‐large datasets acquired only from low‐diversity samples and require exquisite quantitative data.

Most metaproteomics studies view the sampled microbial community as a static system, but it may be more dynamic than currently assumed. Isotope‐labelled compounds delivered to a microbiota during an experiment can be differentiated by mass spectrometry by examining their metabolites or the proteins themselves. This approach provides insightful results pinpointing the active components of the system studied (Seifert et al., 2012; Starke et al., 2016). Identifying specific protein biomarkers or the overall proteome profile can also help characterize the state of microbiome components. Dead material (i.e., necromass) can be distinguished from viable but non‐culturable cells and active microorganisms, like spores and vegetative cells. These different states can thus be readily distinguished and quantified, as recently illustrated (Mappa et al., 2021). Targeted metaproteomics analysis of such markers can, in principle, be developed. Whether these markers can be generalized for a wide range of organisms or will be directly accessible by discovery‐oriented metaproteomics requires further exploration. Detailed snapshots of the metaproteome of any microbial system over time or under different conditions can be readily obtained. As protein components may be located in subcellular compartments or outside cells, this functional information cannot be simply predicted from the genome, even with the best algorithms (Douglas et al., 2020). I speculate that meta‐analyses of metaproteomics results, together with other experimental methodologies measuring specific protein activities (Pudlo et al., 2022), could help improve the predictors currently widely used to forecast microbial functions from metagenome sequences. Last, it goes without saying that projects combining metaproteomics and metabolomics will be commonplace in the future in order to refine our knowledge of the metabolism of microbiota.

## POSSIBLE FUTURE APPLICATIONS OVER THE NEXT DECADE

**FIGURE 4:**
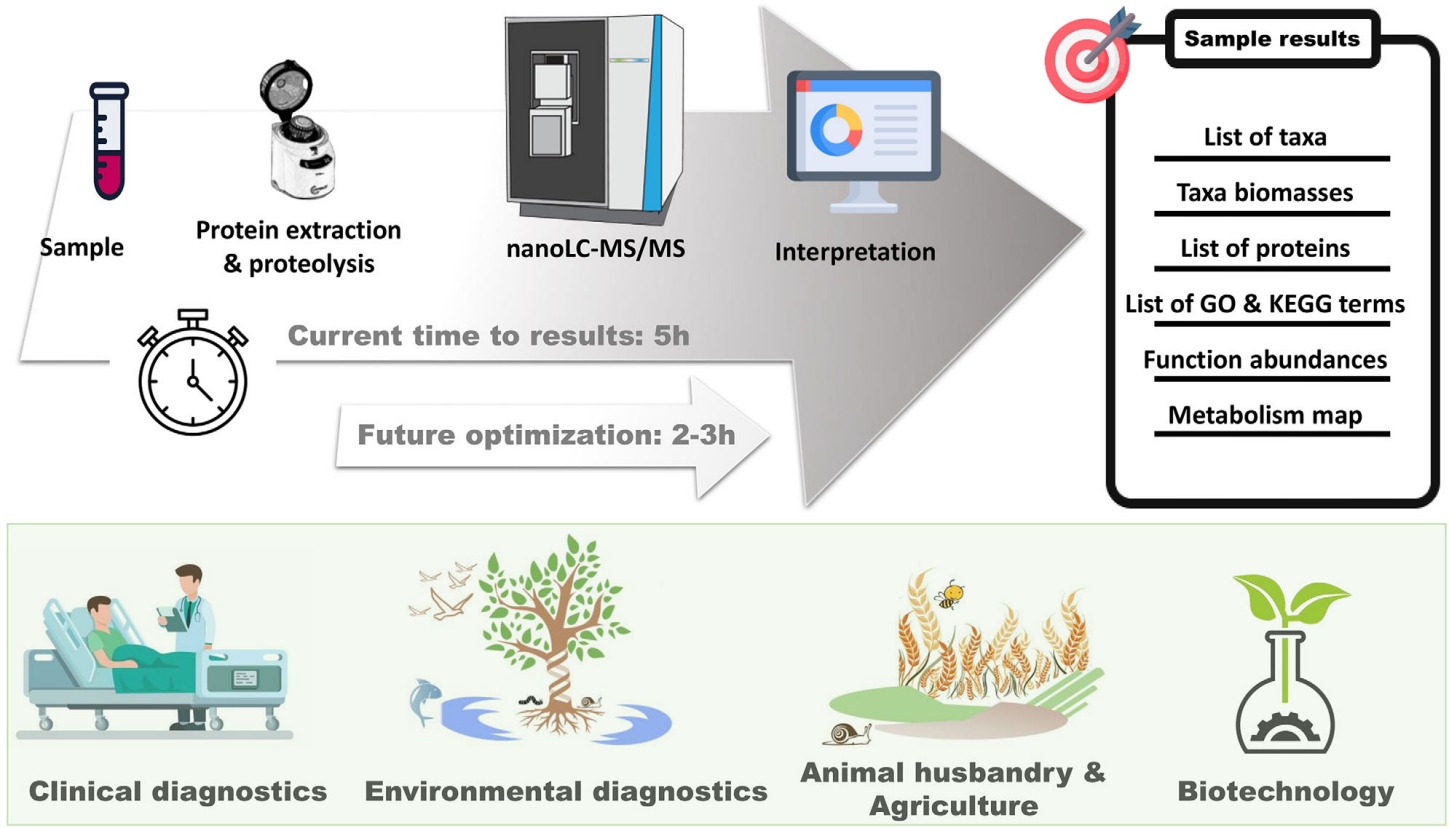
Clinical and environmental diagnostics by metaproteomics, timelines and applications. A sample‐to‐result timeline is proposed based on previously published results (Hardouin et al., 2022), along with likely optimizations over the next decade. The fields of application of metaproteomics for diagnosis or routine analysis are schematized.

High‐resolution tandem mass spectrometry is a rapid technique. Typically, 1 or 2 h of mass spectrometry per sample are sufficient to discover notable changes in the structure or functions of microbial communities. Sample preparation can also be reduced to less than an hour (Hayoun et al., 2020), except for challenging samples such as soils (Herruzo‐Ruiz et al., 2021; Keiblinger et al., 2012) or for more specific sample preparations (Salvato et al., 2022). An automated, refined interpretation of metaproteomics results based on cascade searches applicable to any sample can be completed within 2 h (Hardouin et al., 2022). Thus, currently, the time between sampling and result could be less than 5 h (Figure 4) compared to 24 h workflow proposed 3 years ago (Heyer et al., 2019), leaving plenty of leeways to optimize the various stages of the procedure and obtain results even faster, thanks to improved protocols and greater computing power. Additionally, labelling samples to allow multiplex analysis could be attractive to reduce overall mass spectrometry time and the associated costs. Therefore, projects involving metaproteomics are likely to set much more ambitious goals in the coming years, leading to a significant increase in the number of samples handled, analysed, and compared. Accordingly, metaproteomists should strive to improve the robustness of the analytical pipeline and benchmark their protocols through multi‐centre evaluations.

Metaproteomics provides a detailed list of taxa present in a sample, a quantitative view of these taxa at different taxonomic levels, functional information such as the production of toxins, antibiotic resistances, and other microbial virulence markers, as well as host markers indicative of inflammatory status or defence efficiency, and the abundance of these functions. This information could also serve to obtain a quick modelled metabolism of the whole sample. All this information could be used clinically for diagnosis and a more personalized medicine (Figure 4). Faecal, oral and pulmonary metaproteomics have already proven to be very informative (Hardouin et al., 2021; Henry et al., 2022; Young et al., 2015). The efforts required to introduce and validate the approach for routine use in clinical diagnostics will be enormous, but the wealth of information to be obtained and the expected health benefits are greater still.

The same can be said for animal health and environmental monitoring. Using mass spectrometry to monitor water quality, soil diversity, overall biodiversity, or ecosystem changes could be as easy and even faster than sequencing Environmental DNA (eDNA). For example, metaproteomics could be used to verify faecal samples from endangered animals to establish their diet and health status. Moreover, metaproteomics could be advantageously applied to improve animal breeding and agricultural practices (Figure 4). Due to global warming, food production will be one of the most significant challenges humanity has to face in the following decades. Consequently, agricultural practices must be adapted to mitigate the effects of climate change. Regular monitoring of soil microorganisms could help farmers to select cultivable plants and probiotic biostimulants based on local soil potential, finding microbiome‐based alternatives to chemical fertilizers or pesticides, and preserving water resources. Metaproteomics is already used to link rumen microbial function to ruminant productivity traits (Andersen et al., 2021). Finally, metaproteomics should help to optimize biotechnological products or processes based on consortia of microorganisms as exemplified by pioneering works on microbial communities of anaerobic digestion plants (Heyer et al., 2020).

Recent studies assessing microbiota biobanking (Zhang et al., 2022) or the functional effects of sweeteners on ex vivo human gut microbiome models (Sun et al., 2022) are paving the way for more systematic use of metaproteomics in a large number of directions. In my opinion, functional microbiome monitoring as a routine analysis, especially for more personalized medicine, is within reach.

Meeting the daunting challenges and applying the metaproteomics presented in this crystal ball article will be possible thanks to the energy and the will of all the members of the very dynamic metaproteomics community and the many microbiome experts willing to get functional insights into their biological systems (Van Den Bossche, Arntzen, et al., 2021). Making at least some of these ideas a reality in the next few years would be rewarding for all of us.

## CONFLICT OF INTEREST

The author has declared no conflict of interest.
